# Leukocyte Profiles in Breeding Female and Pup Southern Elephant Seals (*Mirounga leonina*) in Relation to Lactation and Body Condition

**DOI:** 10.3390/ani16203304

**Published:** 2026-10-20

**Authors:** Pablo Descayrac, Coraline Bichet, Jérôme Badaut, Baptiste Picard, Christophe Guinet, Jordi López-Ramon, Victor Lizana, Caroline Gilbert

**Affiliations:** 1 Servicio de Análisis, Investigación, Gestión de Animales Silvestres (SAIGAS), Facultad de Veterinaria, Universidad Cardenal Herrera-CEU, CEU Universities, 46115 Alfara del Patriarca, Spain; pdescayrac82@orange.fr (P.D.); jordi.lopez1@uchceu.es (J.L.-R.); 2 Centre d’Etudes Biologiques de Chizé, UMR7372 La Rochelle Université-CNRS, 79360 Villiers en Bois, France; coraline.bichet@univ-lr.fr (C.B.); jerome.badaut@cebc.cnrs.fr (J.B.); baptiste.picard@cebc.cnrs.fr (B.P.); christophe.guinet@cebc.cnrs.fr (C.G.); 3 Wildlife Ecology & Health Group (WE&H), Universitat Autònoma de Barcelona (UAB), 08193 Barcelona, Spain; 4 Laboratoire MECADEV, UMR 7179, CNRS/MNHN, Ecole Nationale Vétérinaire d’Alfort, 7 Avenue du Général de Gaulle, 94704 Maisons-Alfort, France

**Keywords:** pinnipeds, phocids, leukocyte concentration, leukocyte profiles, immune system, lactation, body condition

## Abstract

This study analyzed blood smears from southern elephant seal (*Mirounga leonina*) adult females and pups. White blood cell profiles (total WBC count (WBC/10^4^RBC) and the relative percentages (%) of neutrophiles, lymphocytes, monocytes, eosinophils, and basophils, as well as the neutrophil-to-lymphocyte ratio (N/L ratio)) were compared between pups and adult females, according to sex and lactation phase (beginning and end of lactation). Additionally, relationships between WBC variables and body condition were examined, as well as those between maternal WBC variables and their pups’ body condition. Adult females had more neutrophils and higher N/L ratios, but fewer lymphocytes, compared to pups. Between the beginning and the end of lactation, WBC total count increased in adult females. In females at end of lactation, lymphocytes increased with body condition while neutrophils decreased. No relationships were found between pups’ WBC variables and their body condition, or between maternal WBC variables and their pups’ body condition. In pups, no differences were seen between male and female in WBC variables. This study provides valuable insight into the immune compartment of this species. More studies are needed to improve our understanding of the physiology of southern elephant seals and its potential fitness consequences.

## Simple Summary

This research explored the changes in the immune status of southern elephant seal females and pups, from pup birth to pup weaning, by measuring different types of immunity cells, such as neutrophils, lymphocytes, and monocytes. The study looked at how these cells varied based on age, sex, and the nursing stage, and whether they were linked to the animal’s physical condition. Adult females had higher levels of neutrophils, but lower levels of lymphocytes compared to pups. Pups showed no differences between males and females in their immune cell levels. By the end of the nursing period, females had increased total white blood cells, and their lymphocyte levels were positively related to body condition, while higher neutrophils suggested poorer condition. However, no clear link was found between the immune health of mothers and their pups, or between the pups’ immune health and their physical condition. These findings provide insight into the health of this species, offering important data to help understand how they cope with challenges in their environment, which may guide future conservation efforts.

## 1. Introduction

Southern elephant seals (*Mirounga leonina*) are the largest existing pinnipeds [1], exhibiting a circumpolar distribution [2]. They spend most of their time foraging at sea, coming ashore only for brief, terrestrial breeding, lactating and molting periods during which they undergo complete fasting [3,4]. Breeding females give birth to a single pup, nursing it for approximately three weeks exclusively from their stored blubber reserves [5,6]. Consequently, the size of the pups at weaning and their subsequent survival chances depend on the mother’s ability to find food and store reserves during gestation [6]. This maternal investment is exceptionally taxing, with mothers losing an average of 35% of their initial body mass during lactation [7]. This energetic drain and substantial tissue mobilization during absolute fasting acts as a severe metabolic and physiological stressor for the mother [8,9]. In vertebrates, such intensive resource allocations and physiological strains are known to alter circulating leukocyte profiles [10]. Therefore, the lactation period represents a critical physiological window for investigating how extreme maternal investment and nutritional constraints influence baseline cellular immune compartments in both mothers and developing pups.

Assessments of cellular immune components from blood smears provide valuable insights into individual physiological states [11]. In wildlife health surveys, total and differential white blood cell (WBC) counts (specifically focusing on phagocytic neutrophils, adaptive lymphocytes, and monocytes) are widely used to monitor responses to energetic and environmental constraints [10,12,13]. In particular, the neutrophil-to-lymphocyte (N/L) ratio is often employed as an indicator of physiological stress and homeostatic load in vertebrates, as glucocorticoid dynamics can significantly alter these circulating proportions [10]. However, circulating leukocyte profiles are influenced by a wide array of overlapping physiological processes, including reproductive status, nutritional state, and metabolic shifts [14,15]. Therefore, variations in these cellular proportions should be interpreted as complex physiological responses rather than direct or specific measures of absolute immune competence or overall health status.

WBC counts from blood smears are among the most widely used techniques in veterinary, biological, and ecological research to assess cellular immune response. Leukocyte differentials have been extensively used to evaluate immune function in pinnipeds and other mammals [14,16,17]. Blood smear analysis is particularly well suited to the demanding conditions of polar research stations, as it does not require expensive equipment and allows for easy sample collection, preparation, and preservation in the field [18].

Information on the health status of southern elephant seals, and of Antarctic wildlife in general, is scarce and crucial for assessing their ability to respond to pathogens, especially in the current context of global changes, which lead to the introduction, redistribution, or increased virulence of pathogens [19,20], such as the H5N1 virus of the highly pathogenic avian influenza, which has reached the Antarctic continent and the sub-Antarctic islands, leading to a high mortality in elephant seals since October 2024 in the Southern Indian Ocean Islands [21]. Although the present study does not directly assess specific viral susceptibility or immune responses to infection, establishing these baseline leukocyte profiles during critical life-history stages is essential in order to provide reference standards for future health monitoring and population screening.

To address these knowledge gaps, this study was designed with four primary objectives: (1) to compare baseline WBC profiles between breeding adult females and pups; (2) to evaluate longitudinal changes in maternal leukocyte parameters between the beginning and the end of lactation; (3) to assess potential differences in leukocyte variables between male and female pups at weaning; and (4) to investigate the relationships between leukocyte profiles and body condition indexes (BCI), including potential links between maternal immune parameters and their pups’ body condition.

In line with these objectives, we hypothesized that: (i) adult females would display higher baseline markers of innate immunity and physiological load (such as a higher N/L ratio) compared to developing pups, due to age-class variations; (ii) maternal leukocyte profiles would shift across lactation as a consequence of the severe physiological strain and nutritional constraints of absolute fasting; (iii) no marked differences in WBC profiles would be found between male and female pups prior to sexual maturity; and (iv) poorer maternal body condition at the end of lactation would be associated with a shift toward a stress-related leukocyte pattern (neutrophilia and lymphopenia), whereas pup body condition would reflect their investment reserves independently of these maternal baseline traits.

## 2. Materials and Methods

### 2.1. Study Area

Located in the Southern Indian Ocean, the Kerguelen Islands host a large and diverse community of seabirds and marine mammals and are home to the largest or second-largest population of southern elephant seals [22], with nearly 348,000 individuals [23].

### 2.2. Sampling and Data Collection

Blood and biometric samples were collected from a total of 28 unique adult females and a pool of 32 pups at weaning. To capture both cross-sectional and longitudinal dynamics, the adult female sampling was structured as follows: 10 individual females were sampled twice (at the beginning and at the end of lactation), yielding 20 paired samples. The remaining 18 unique females were captured only once, thereby yielding a single cross-sectional sample each. Consequently, a total pool of 38 adult female blood samples was obtained for age-class comparisons.

Regarding maternal–offspring associations, a total of 29 paired mother–pup data points were available for biometric and immunological correlation analyses. This pool comprised 20 paired records obtained longitudinally from 10 unique pairs sampled twice (at the beginning and at the end of lactation), plus 9 cross-sectional pairs sampled exclusively at weaning. For the wider analysis of pups at weaning (*n* = 32 total), the sample comprised 29 independent pups (average age of 21 days; 29 with recorded sex) who were completely independent of the adult female pool, plus 3 pups belonging to sampled mothers from our main pool. The beginning of lactation was operationally defined in the field by the presence of newborn pups (with a wet umbilical cord and an intact black coat) [24], while the end of lactation was defined by the imminent weaning indicated by the advanced state of molt, body condition and/or permanent absence of the mother from the harem [25,26].

To ensure safe handling, adult animals were chemically immobilized via intramuscular injection with a combination of 1 mg/kg tiletamine—zolazepam (Zoletil 100^®^ Virbac, Carros, France) [27]. Blood was collected from the epidural sinus [15,28,29]. For pups, weight measurements were taken using a dynamometer (precision of 0.5 kg). For adults, weight was taken using a hoist suspended from a tripod. Standard length was measured from the tip of the snout to the end of the tail [22]. The Body Condition Index (BCI) was calculated as mass (M) divided by the square of the standard length (SL):

$$\mathrm{BCI}=\frac{M\left(\mathrm{kg}\right)}{SL^{2}\left(\mathrm{cm}\right)}$$

This specific morphometric index has been previously validated and widely utilized for southern elephant seals to monitor energy reserve fluctuations on land, as it scales appropriately with body mass changes independent of structural size skeleton constraints [30,31,32]. As a maternal investment index, a differential body condition index (dBCI) was calculated for double-sampled females (*n* = 10), using the equation

$$\mathrm{dBCI}=\frac{M_{i}-M_{f}\left(\mathrm{kg}\right)}{SL_{\mathrm{mean}}^{2}\left(\mathrm{cm}\right)}$$

where M*_i_* is the initial mass, M*_f_* is the final mass, and *SL*_mean_ is the mean of both standard-length measurements, considering that structural standard length does not change significantly over a 3-week lactation period.

Morphometric mass-to-length indices have inherent limitations, as they serve as proxy measures of adiposity and cannot mathematically differentiate between the mobilization of fat reserves (blubber) and lean mass tissue or absolute fluid loss during extreme starvation periods [7,8,33,34].

### 2.3. Blood Smears

Immediately upon blood collection, a single drop of fresh blood was smeared onto a clean microscope slide in the field. After air-drying under ambient field conditions, slides were transported back to the research station, where they were fixed using absolute methanol for 5 min. Staining was performed using a 1/20 Giemsa solution (diluted in a pH 7 buffer solution) for 40 min [35]. The stained smears were rinsed with a pH 7 buffer solution and dried for 24 h.

### 2.4. Microscopic Evaluation of the Blood Smears

To determine the leukocyte count, the protocol described by Menéndez-Blázquez and collaborators [36] was followed. This process begins by selecting a homogeneous monolayer area of red blood cells that provides a clear view of the white blood cells. Areas were chosen where erythrocytes filled the entire microscope field at 400× magnification without overlapping. Three fields within this area of the smear were randomly selected, and the number of erythrocytes observed in each field was counted, to approximate the average number of erythrocytes per field. Then, the number of fields needed to observe an estimated total of 10^4^ erythrocytes was calculated. Based on this, leukocytes were counted across this number of fields, obtaining the leukocyte total count per 10^4^ erythrocytes. Moreover, the leukocyte differential counts were determined for the first 100 leukocytes counted on a single smear per individual by classifying these leukocytes as neutrophils, lymphocytes, monocytes, eosinophils, or basophils [14,17]. We explicitly acknowledge that while counting 100 leukocytes per slide is an optimized and practical approach for remote wildlife field studies, it introduces potential sampling variation, particularly for relatively rare cell types such as basophils and eosinophils, whose minor proportions should be interpreted as general trends. Microscopic observations were performed using a Leica DM750 microscope at 1000× magnification under immersion oil. To ensure methodological consistency and minimize potential observer bias, all microscopic evaluations and cell counts were performed blindly regarding individual animal identity, and by a single researcher. As an internal quality-control measure, duplicate counts were conducted on a random subset of individual slides to verify intra-observer consistency.

### 2.5. Statistical Analysis

To account for individual-level dependence within the longitudinal subset of 10 repeatedly monitored females and mother–pup pairs, data over time were strictly analyzed using paired *t*-tests or Wilcoxon signed-rank tests exclusively on this isolated dataset, whereas pooled cross-sectional analyses (*n* = 38) were treated independently.

The seven WBC variables measured (WBC total count, neutrophil, lymphocyte, monocyte, eosinophil, and basophil levels, N/L ratio) were compared between adult females (*n* = 38) and pups (*n* = 32) using parametric (Student’s *t*-test) or non-parametric (Mann–Whitney *U* test) tests, depending on data distribution. Normality was evaluated using the Shapiro–Wilk test, and homogeneity of variances was confirmed with Levene’s test. Normally distributed data with equal variances were analyzed using independent-samples *t*-tests, while non-normal or heteroscedastic data were compared using the Mann–Whitney *U* test. Results are presented as mean ± standard deviation (SD) for parametric data and median (interquartile range, IQR) for non-parametric data. Effect sizes were calculated as Cohen’s *d* for *t*-tests and rank-biserial correlation (*r_c_*) for Mann–Whitney tests. To assess changes in hematological parameters over time during lactation, paired *t*-tests (for normally distributed data) or Wilcoxon signed-rank tests (for non-normal data) were performed using our double-sampled adult females data (at beginning and end of lactation, *n* = 10). In order to investigate the relationship between body condition and WBC variables at the beginning and end of lactation, Pearson correlation coefficients were calculated between BCI and hematological parameters, at both periods (*n* = 10), when normality was confirmed using the Shapiro–Wilk test (*p* > 0.05). Spearman correlation was used for non-normal variables. The same relationships were investigated for our total adult female samples (*n* = 38). WBC parameters were compared between male (*n* = 11) and female (*n* = 18) pups, using parametric (Student’s *t*-test) or non-parametric (Mann–Whitney *U* test) tests, depending on data distribution. Normality was evaluated using the Shapiro–Wilk test, and homogeneity of variances was confirmed with Levene’s test. Relationships between pups BCI and their mother’s WBC parameter (for total adult female data linked to a biometric sampled pup, *n* = 29) were evaluated using Spearman correlation according to data distribution. Relationships between pups’ BCI and their WBC parameters (for all blood-sampled pups, *n* = 29, which do not all correspond with the precedent analysis pool: *n* = 29) were evaluated using Pearson and Spearman correlation according to data distribution. Because several leukocyte parameters were examined simultaneously, these analyses should be considered exploratory. No alpha-level adjustments for multiple comparisons were applied to the dataset, and a standard significance threshold of alpha = 0.05 was maintained throughout. All analyses were performed using jamovi (Version 2.7.5.0) [37].

## 3. Results

### 3.1. Biometric Measurements

In the overall dataset, a total of 38 independent measurements corresponding to 28 adult females were evaluated. On average, adult females had a standard body length of 242.00 ± 18.48 cm, a net mass of 366.24 ± 89.76 kg and a BCI of 0.62(±0.12) × 10^−2^ kg/cm^2^. Within these records, the longitudinally monitored subset (*n* = 10 unique individuals, providing 20 paired measurements) reflected statistical shifts over the nursing period. At the beginning of lactation, maternal measurements exhibited a mean body mass of 432.60 ± 81.25 kg and a BCI of 0.74(±0.07) × 10^−2^ kg/cm^2^. By the end of lactation, mean body mass dropped to 293.65 ± 49.23 kg and BCI decreased to 0.52(± 0.04) × 10^−2^ kg/cm^2^. Paired comparisons showed that both the absolute mass loss (138.95 ± 37.99 kg *t*(9) = 11.57, *p* < 0.001) and subsequent decline in BCI (*t*(9) = 9.82, *p* ≤ 0.001) were significant. This mobilization of maternal reserves resulted in a mean differential condition index (dBCI) (as maternal investment index) of 1.19(±0.30) × 10^−4^ kg/cm^2^.

For the offspring component, statistical analyses were restricted to the independent pups with a complete biometric and sex record (*n* = 29). At weaning, these pups exhibited a mean standard body length of 135.00 ± 7.68 cm, a net body mass of 112.00 ± 21.30 kg, and a BCI of (0.61 ± 0.07) × 10^−2^ kg/cm^2^. When analyzing paired maternal–pup records (*n* = 29 available pairs) at earlier lactation stages, the corresponding pups showed a mean standard length of 125.00 ± 15.20 cm, a body mass of 69.90 ± 39.00 kg, and a BCI of (0.42 ± 0.16) × 10^−2^ kg/cm^2^.

### 3.2. Comparison Between the WBC Profiles of Pups and Adult Females

Descriptive statistics and group comparisons for hematological parameters are summarized in Table 1 and Table 2 and Figure 1. Significant differences were observed between adult female blood samples (*n* = 38) and pup blood samples (*n* = 32). Neutrophil relative percentages were significantly higher in adult maternal samples than in pups (*t*(68) = 5.06, *p* < 0.001, *d* = 1.21), which consequently led to a significantly elevated N/L ratio (*U* = 273, *p* < 0.001, *r_c_* = 0.55). Conversely, pups exhibited a significantly higher relative percentage of both lymphocytes (*t*(68) = −4.11, *p* < 0.001, *d* = −0.99) and monocytes (*U* = 370, *p* = 0.005, *r_c_* = −0.39), compared to adult females. No statistically significant differences were detected in total WBC count, relative eosinophils, or relative basophils (*p* > 0.05 for all comparisons). The Shapiro–Wilk test results and Levene’s test results are available in Appendix A, Table A1 and Table A2.

### 3.3. Comparison Between Beginning and End of Lactation in Adult Females’ WBC Profiles

Descriptive statistics for the WBC profiles of adult females at the beginning and at the end of lactation are summarized in Figure 2 and in Appendix A, Table A3. Paired comparison results between these parameters are shown in Appendix A, Table A4. Shapiro–Wilk test results are available in Appendix A, Table A5. Significant differences were observed in WBC total count (*t*(9) = −2.513, *p* = 0.033, d = −0.795), which was higher at the end of lactation. No other significant differences were detected between hematological parameters (*p* > 0.05).

### 3.4. Relationship Between WBC Parameters and Biometric Variables in Adult Females

Biometric variables for the 10 longitudinally monitored adult females are provided in Appendix A, Table A6. Corresponding normality tests (Shapiro–Wilk test) for maternal morphometrics and hematological variables at both the beginning and the end of lactation are available in Appendix A, Table A7, Table A8 and Table A9.

No significant correlations were observed between maternal leukocyte parameters and the body condition index at the beginning of lactation (BCI; all *p* > 0.05). As expected, due to mathematical dependency, strong internal correlations were present among immune cell populations and their derived ratios at this initial stage (lymphocytes vs. neutrophils: *r*(8) = −0.961, *p* < 0.001; N/L ratio vs. neutrophils: *r*(8) = 0.783, *p* = 0.007; and N/L ratio vs. lymphocytes: *r*(8) = −0.849, *p* = 0.002) (Figure 3).

Regarding the end of lactation, the relationships between maternal leukocyte frequencies and energy reserves were evaluated. Neutrophils were negatively correlated with maternal BCI (*r*(8) = −0.640, 95% CI [−0.905, −0.017], *p* = 0.046, Figure 4A), whereas lymphocytes were positively correlated (*r*(8) = 0.669, 95% CI [0.068, 0.914], *p* = 0.034, Figure 4B). Additionally, the N/L ratio was negatively correlated with adult female BCI (*r_s_*(8) = −0.721, 95% CI [−0.932, −0.146], *p* = 0.024, Figure 4C). Other physiological or morphometric correlations did not reach statistical significance in this sample (Table 3), with internal blood-smear parameters continuing to reflect their expected mathematical constraints (lymphocytes vs. neutrophils: *r_s_*(8) = −0.946, 95% CI [−0.988, −0.744], *p* < 0.001 (Figure 5A); N/L ratio vs. neutrophils: *r_s_*(8) = 0.939, 95% CI [−988, −0.774], *p* < 0.001; N/L ratio vs. lymphocytes: *r_s_ (8)* = −0.997, 95%CI [−0.999, −0.986], *p* < 0.001*).* Additionally, a significant positive correlation was observed between basophils and monocytes (Figure 5B). Pearson (for normally distributed data) and Spearman correlation coefficients for the relationships between WBC and biometric variables at different lactation stages are presented in Table 3.

When pooling all adult female samples (*n* = 38), regardless of lactation stage, no significant correlations were detected between body condition index (BCI) and WBC parameters (all *p* > 0.05; Spearman correlation coefficients are available in Appendix A, Table A10 and the corresponding normality tests in Appendix A, Table A11).

No significant correlations were seen between differential body condition index (dBCI) (as maternal investment) and WBC parameters at end of lactation in our double-sampled adult females (*n* = 10) (all *p* > 0.05; corresponding normality test and correlation coefficients are available in Table A12 and Table A13, Appendix A).

### 3.5. Comparison Between Male and Female Pups

No significant differences were observed between male and female pups (*p* > 0.05 for all comparisons). The descriptive statistics are summarized in Table 4 and Figure 6, and group comparisons for hematological parameters are summarized in Table A16. Shapiro–Wilk test results and the Levene’s test results are available in Table A14 and Table A15, Appendix A.

### 3.6. Relations Between Pup’s BCI and Their Mother’s WBC Variables

There were no significant correlations between pup’s BCI and their mother’s WBC variables (*n* = 29), (all *p* > 0.05). Pearson and Spearman correlation coefficients are available in Appendix A, Table A19. Corresponding normality analysis (Shapiro–Wilk) is available in Appendix A, Table A17.

### 3.7. Relations Between Pup’s BCI and Their Own WBC Variables

There were no significant correlations between pup’s BCI and their own WBC variables (*n* = 29), (all *p* > 0.05). Pearson and Spearman correlation coefficients are available in Appendix, Table A19. Corresponding normality analysis (Shapiro–Wilk) is available in Appendix A, Table A18.

## 4. Discussion

To our knowledge, there is only one previous study on the WBC profile of the southern elephant seal. Overall, the results of that study mostly align with ours, differing essentially in the case of eosinophils (see Table 5). While this study reports 0.63%, the study by Melrose and colleagues [38] shows this value increasing to 15%. Several factors may explain this difference, such as variability due to the small number of animals studied, as this species lives in remote areas and requires specialized logistics. Alternatively, this variance could putatively be explained by differences in parasitism between the two populations, with eosinophils playing a primary role in the immune response against parasites [39]. While the individuals sampled by Melrose et al. could have been more heavily parasitized than the Kerguelen population studied here, this hypothesis remains speculative for our study population and should be interpreted strictly as a tentative explanation rather than a definitive conclusion [39].

In northern elephant seals (*Mirounga angustirostris*) during the reproductive period, leukocyte differentials obtained through automated cell counters are relatively similar to ours, except for eosinophil levels, which were again higher (2% and 6.52% versus 0.63%) [15,29]. When compared with other species, common seals (*Phoca vitulina*) and California sea lions (*Zalophus californianus*) have been reported to have lower neutrophil and higher lymphocyte values [29]. These comparisons can be observed in Table 5. The WBC total count in our study (64.03 ± 50.5 WBC/10^4^RBC in females and 81.47 ± 48.8 WBC/10^4^RBC in pups) was significantly higher than previously measured in southern elephant seals (34.40 ± 3.92 WBC/10^4^RBC) and other pinniped species: 32.20 ± 4.29 GB/10^4^RBC in crabeater seals (*Lobodon carcinophaga*), 38.00 ± 5.46 WBC/10^4^RBC in Weddell seals (*Leptonychotes weddellii*) [40], and 23.00 ± 2.80 WBC/10^4^RBC in leopard seals (*Hydrurga leptonyx*) [41]. Rather than looking for a single physiological explanation for these inter-species variations, the distinct patterns should be viewed objectively as a combination of biological traits (such as differences in age, reproductive stage, or fasting duration), and substantial methodological heterogeneity, including the use of automated cell counters versus manual blood smears and the lack of standardized measurement units across research groups. Comparing total WBC counts studies is difficult due to these differences in measurement units.

Our results have shown that pups had significantly lower neutrophil counts and a lower N/L ratio compared to adult females, while lymphocyte counts were higher. This result is a classic pattern in mammalian hematology. In most species, young animals exhibit physiological lymphocytosis (high lymphocyte count) which decreases with age, while the number of neutrophils tends to increase or stabilize. This reflects the intense activity of the thymus and the development of the adaptive immune system in young individuals (clonal expansion of naive lymphocytes). In adults, the immune system evolves towards greater dependence on innate immunity (neutrophils) and an immune ‘memory’ that requires fewer circulating lymphocytes in absolute terms [42,43]. However, interpreting these baseline leukocyte differences between cohorts requires caution. In this study, age and reproductive status are inherently confounded, as developing pups were compared directly against adult breeding females undergoing a critical life-history stage. Consequently, the observed immunological divergence cannot be attributed to age-related maturation alone. Instead, these distinct profiles likely reflect a multifaceted interplay of developmental stage, maternal reproductive state, prolonged fasting, and the severe energetic expenditure experienced by lactating females A major study on bottlenose dolphins (*Tursiops truncatus*) confirms that absolute lymphocyte counts decrease significantly with age, being highest in juveniles [44].

In the harbor seal (*Phoca vitulina*), as observed in our study between pups and adult females, neutrophil counts increased with age, while lymphocytes significantly decreased [45]. Harbor seal pups have markedly lower WBC total count than their mothers at birth, but they reach similar values by two days of age [46]. This increase in WBC total count is due to the rising number of neutrophils, lymphocytes, and, to a lesser extent, monocytes. However, the overall increase in lymphocytes and monocytes throughout lactation appears to be the main factor contributing to the rise in total WBC count, as the percentage of neutrophils decreases later in the lactation period [46]. Although harbor seal mothers appeared to have slightly higher eosinophil and basophil counts compared to their pups, no significant differences were found for these cell types in our study, which reinforces the need to treat potential drivers like differential parasitism as speculative trends rather than established baselines [14]. Similarly to our findings, captive juvenile harbor seals exhibited lower neutrophil counts, but higher lymphocyte and monocyte levels compared to wild adults. They also showed reduced total WBC and eosinophil counts [17].

In contrast to what was observed in the harbor seal, in Steller sea lions (*Eumetopias jubatus*) and gray seals (*Halichoerus grypus*), higher WBC counts have been documented in pups and juveniles, compared to adults [17,47]. Unlike our results, neutrophil counts decreased with age in Steller sea lion pups, along with a decline in the N/L ratio due to the drop in neutrophils [17]. Steller sea lion pups aged 6 to 10 months had total white blood cell counts lower than those of juveniles (1 to 3 years) [48] and lower than those of younger pups (5 to 38 days old) [17]. We deduce that these animals exhibit a high white blood cell count at birth, followed by a postnatal decline, which increases again in juveniles, and at a certain age, decreases once more.

As in this study, no significant effects of sex on WBC total count and formulation have been observed in Steller sea lion pups (*Eumetopias jubatus*) [17] or in leopard seals, Weddell seals, gray seals, or northern elephant seals [40,41,47,49,50]. Higher neutrophil and monocyte levels in males and higher lymphocyte levels in females have only occasionally been observed in harbor seals (*Phoca vitulina*) [51].

The results show a higher WBC count in females at the end of lactation compared to the beginning. Rather than representing an established causal relationship, this empirical observation may reflect a complex, multi-factorial physiological adjustment to the energetic demands of nursing. In elephant seals, lactation is a period of intense fasting for the mother. However, even in species that do not fast, lactation is considered a major ‘metabolic stress’ and a pro-inflammatory state. The observed increase in WBC at the end of lactation may reflect the mobilization of body reserves and a systemic response to energy depletion. Hypothetically, the mobilization of lipid reserves (lipolysis) necessary to support milk production might induce a low-grade systemic inflammatory response, which could contribute to shifting leukocyte proportions [52]. The intense metabolic activity required for milk synthesis is a potential pathway for generating reactive oxygen species (ROS). This oxidative stress might activate leukocytes and can increase circulating numbers in response to tissue inflammation [53]. Additionally, the principal active endocrine factor during lactation, the prolactin hormone, represents another plausible mechanism that may modulate WBC count, especially by stimulating the activation and proliferation of B-lymphocytes [54,55]. Previous studies on terrestrial species, such as the American badger (*Taxidea taxus*), have shown that food deprivation can lead to an elevated WBC count [56]. In harbor seals, no significant variation in WBC total count has been observed in mothers between the beginning and end of lactation or in pups during post-lactation fasting. Their lactation period is relatively equivalent to that of southern elephant seals. No changes in leukocyte subpopulations were observed, either [14]. Contrary to our findings, a decrease in WBC total count throughout lactation has been reported in Australian sea lion (*Neophoca cinerea*) females. This species does not fast completely during lactation, which could suggest that this effect is mainly due to food deprivation [14].

WBC total count was not significantly related to body condition. In many mammalian species, the total number of circulating leukocytes increases with body weight in adults [57,58,59]. In male harbor seals, higher WBC total counts and neutrophil counts were observed after years of abundant Clupeidae (energy-rich prey) [60]. In other pinniped species (harbor seals and Weddell seals), no influence of body condition on WBC profiles was observed [17,49]. Goldstein et al. [47] associated high WBC total counts with weight gain in juvenile northern elephant seals.

In the southern elephant seals sampled in this study, significant relationships were found between changes in the numbers of lymphocytes (increasing) and neutrophils (decreasing) and the BCI in females at the end of lactation. As seen in Figure 4A,B, a higher BCI corresponded to a higher lymphocyte count and a lower neutrophil count. Conversely, a female with a low BCI at the end of lactation would have a low lymphocyte count and a high neutrophil count. This profile (neutrophilia + lymphopenia) is highly consistent with a generalized physiological stress response, often contextually described in the mammal literature as a ‘stress leukogram’ [61,62]. Given that direct measurements of cortisol or other independent endocrine biomarkers were not included in the present study, this hematological shift must be interpreted cautiously. Rather than implying a verified stress response, this pattern may reflect a complex, multi-factorial response driven by the overlapping physiological strains of prolonged fasting, extensive tissue mobilization (lipolysis), systemic metabolic changes, or underlying tissue inflammation inherent to late lactation, alongside individual physiological variation.

In northern elephant seals, cortisol concentrations increase drastically during the fasting experienced by lactating females, weaned pups, and molting individuals [9,34]. During lactation, a negative correlation has also been observed between cortisol levels and body mass in northern elephant seal pups [63]. In adult males, cortisol levels have also been negatively correlated with body mass during late fasting [64]. Given that glucocorticoid secretion in physiological stress situations causes an increase in circulating neutrophils and a decrease in lymphocytes [65,66], this observed leukogram shift may reflect multifactorial physiological adjustments linked to declining body condition and acute energetic depletion, rather than representing a verified endocrine stress response *per se*.

Stress-induced reductions in circulating lymphocyte numbers are due to the redistribution of lymphocytes from the blood to other body compartments. In response to glucocorticoids, circulating lymphocytes adhere to endothelial cells lining blood vessel walls and subsequently migrate from the circulation to other tissues, such as lymph nodes, spleen, bone marrow, and skin, where they become sequestered [65].

Conversely, glucocorticoids also stimulate the influx of neutrophils into the blood from the bone marrow and reduce the exit of neutrophils from the blood into other compartments [65]. In emaciated northern elephant seals, neutrophilia and lymphopenia have been observed. The same was true for individuals suffering from dermatological diseases during molting (“Scraby molt”) and parasitic infestations [29].

Leopard seals (*Hydrurga leptonyx*) that were considered clinically affected showed higher neutrophil counts and lower lymphocyte counts in sick individuals [41]. However, this pattern is not universal, as body condition in leopard seals has been positively correlated with the N/L ratio [49].

These changes in the leukogram are to the benefit of a more effective innate immunity, as neutrophiles are responsible for the first line of host immune response against invading pathogens, by the means of several mechanisms such as chemotaxis, phagocytosis, release of reactive oxygen species (ROS), granular proteins and proliferation and liberation of cytokines [67]. On the other hand, lymphocytes (B cells; T cells: CD4-positive, CD4/CD8- negative or CD8-positive; and natural killer T cells) are responsible for adaptative immunity, providing an antigen-specific response whose circulating pool appears down-regulated under these physiological conditions [68].

A reconfiguration of the immune system to minimize resource expenditure during periods of physiological stress has been described as a possible interpretation representing a potential adaptative evolutionary strategy [68].

Innate and acquired immune responses differ in both energetic costs and functional efficiency. Acquired immunity entails high developmental costs but is relatively inexpensive to maintain and activate. In contrast, innate immunity has lower developmental costs but incurs higher ongoing energetic demands. From an evolutionary perspective, the balance between these two arms of the immune system is shaped by ecological context. Important nutritional stress, as encountered during the energetically taxing end of lactation in southern elephant seal females, may favor a shift toward innate immune defenses. While less specific, these provide rapid protection against immediate threats [69].

From an evolutionary perspective, while this framework is highly informative, it must be treated with caution as a tentative hypothesis for future research, since static leukocyte profiles cannot confirm functional shifts in immune investment. The observed pattern (neutrophilia with lymphopenia) might tentatively suggest a potential prioritization of innate over adaptive mechanisms under energetic constraints, but our data do not allow for direct conclusions regarding absolute immune competence. Nevertheless, further experimental and physiological evidence, including functional immune assays and cytokine expressions, is strictly required in order to validate whether these circulating shifts translate into true adaptive reallocation strategies under acute energetic constraints.

Several limitations of the present study should be explicitly acknowledged. First, the size of the longitudinally monitored adult female cohort was relatively small (*n* = 10), restricted by the severe logistical and geographic challenges inherent to sampling wild populations in remote sub-Antarctic environments. Consequently, statistical power was constrained, and non-significant trends must be interpreted with caution, as patterns were those observed within the sampled individuals rather than definitive baseline rules for the species. Second, our study relies on a strictly correlational design, which precludes the inference of direct causal relationships between declining body condition and hematological variations. Third, because direct measurements of cortisol, metabolic hormones, tissue-level inflammation, and functional immune performance were not included, the physiological mechanisms proposed, such as glucocorticoid-mediated leukocyte trafficking or adaptive resource reallocation, remain solid working hypotheses that require empirical validation.

In synthesis, the present study provides valuable baseline information on the leukocyte profiles of southern elephant seals (*Mirounga leonina*) at Kerguelen, identifying critical age-related variations and a notable post-lactation shift toward neutrophilia and lymphopenia that correlates with maternal energy depletion. However, while these hematological associations merit further investigation, our data do not establish direct fitness consequences or demonstrate how these immune reconfigurations influence the animals’ capacity to cope with broader environmental challenges. To fully determine the biological and fitness significance of these observed patterns, future research must pivot toward larger longitudinal studies. Such frameworks should ideally combine comprehensive leukocyte profiling with sequential body condition metrics, direct endocrine indicators (such as cortisol), parasitological or pathogen loads, and active functional immune measures. Acknowledging these constraints transparently establishes a clear roadmap for validating the true evolutionary and conservation implications of pinniped immunophysiology.

## 5. Conclusions

The present study establishes a foundational baseline for the total and differential white blood cell (WBC) profiles in breeding southern elephant seal (*Mirounga leonina*) adult females and pups, cohorts for which limited hematological data are currently available. Our results are broadly consistent with previous studies, differing essentially in the case of eosinophil percentages.

The higher lymphocyte frequencies observed in pups alongside higher neutrophil levels in adult females represent a distinct age and class difference rather than a demonstrated longitudinal decrease with age, as these cohorts occupy entirely different developmental and reproductive stages.

These findings highlight a significant post-lactation hematological shift in adult females (characterized by neutrophilia and lymphopenia) that correlates with maternal energy depletion (BCI decline). Rather than establishing a direct causal link to stress, this pattern is consistent with a generalized physiological response to the combined strains of prolonged fasting and reproductive investment. Conversely, no relationships were detected between these immune parameters and biometric data in pups, or between maternal leukocyte profiles and pup biometric variables, providing a robust reference framework for future immunophysiological monitoring in wild pinniped populations.

## Figures and Tables

**Figure 1:**
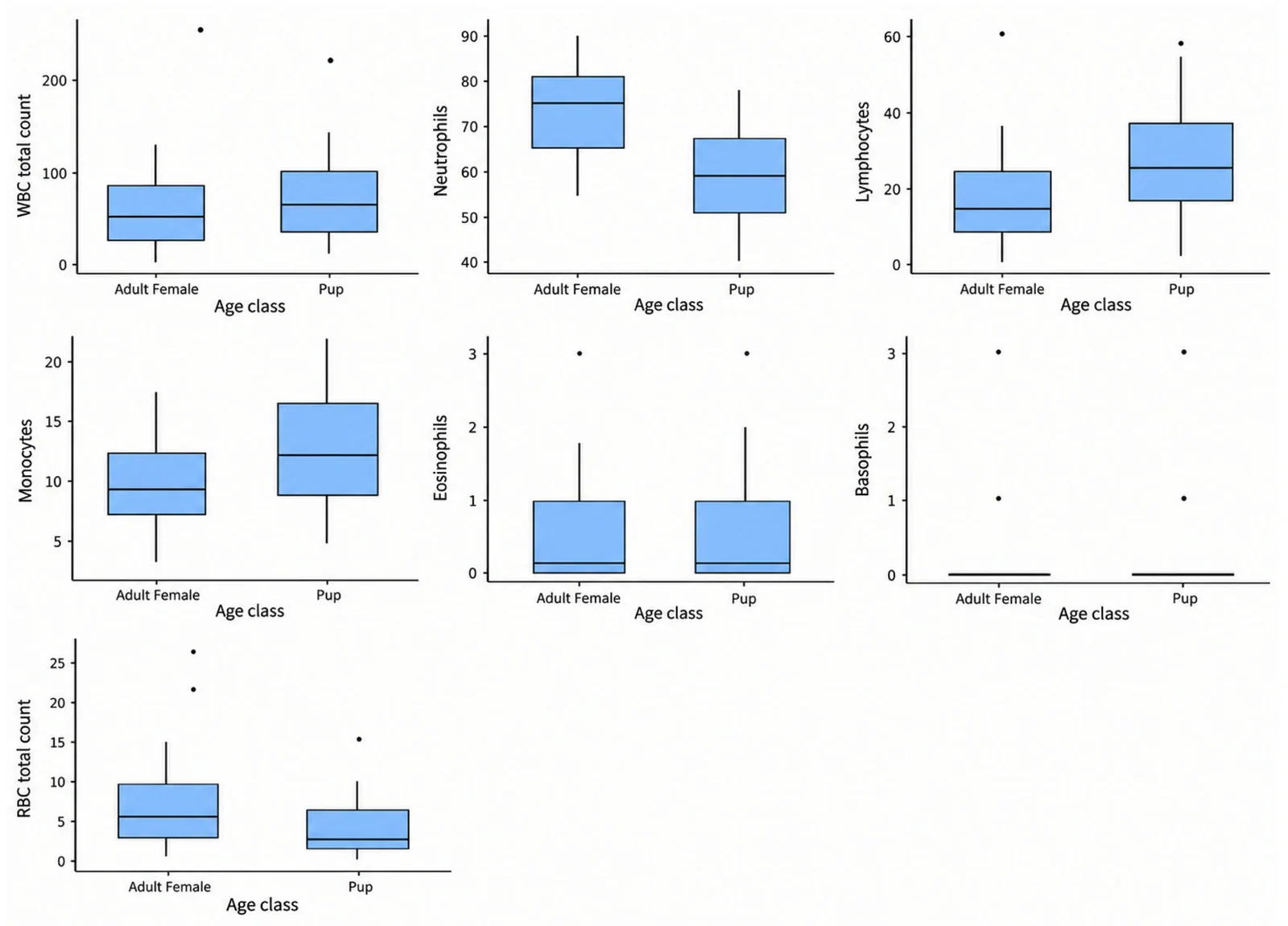
Boxplots for WBC parameters comparison between age classes. Data represent means ± SD for normally distributed variables (Total WBC count, neutrophils, lymphocytes, and monocytes) and medians (with minimum–maximum ranges) for non-normal variables (eosinophils, basophils, and N/L ratio), based on (*n* = 38) adult female samples and (*n* = 32) pup samples.

**Figure 2:**
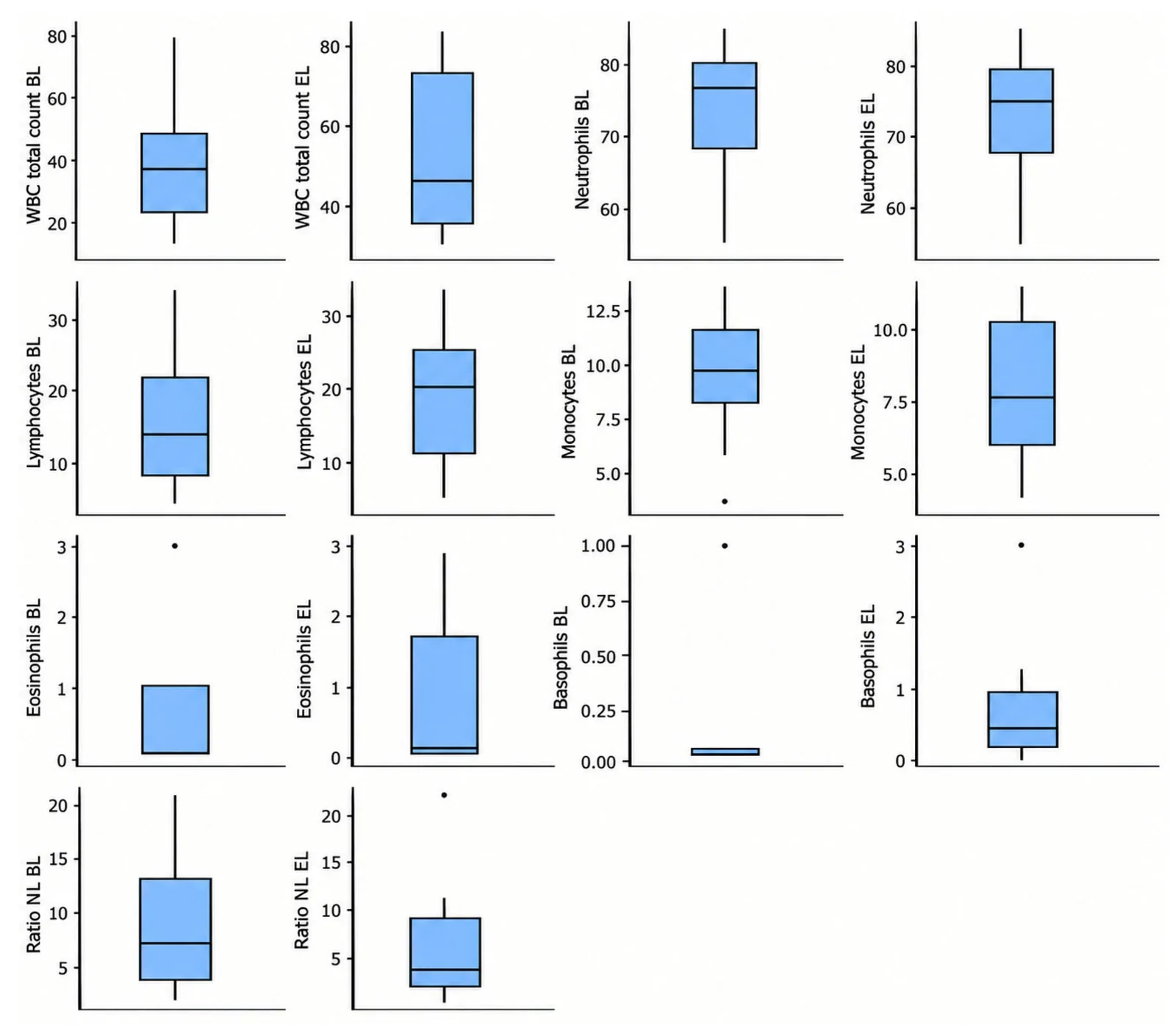
Boxplots for WBC variables in adult females at the beginning (BL) and end (EL) of lactation.

**Figure 3:**
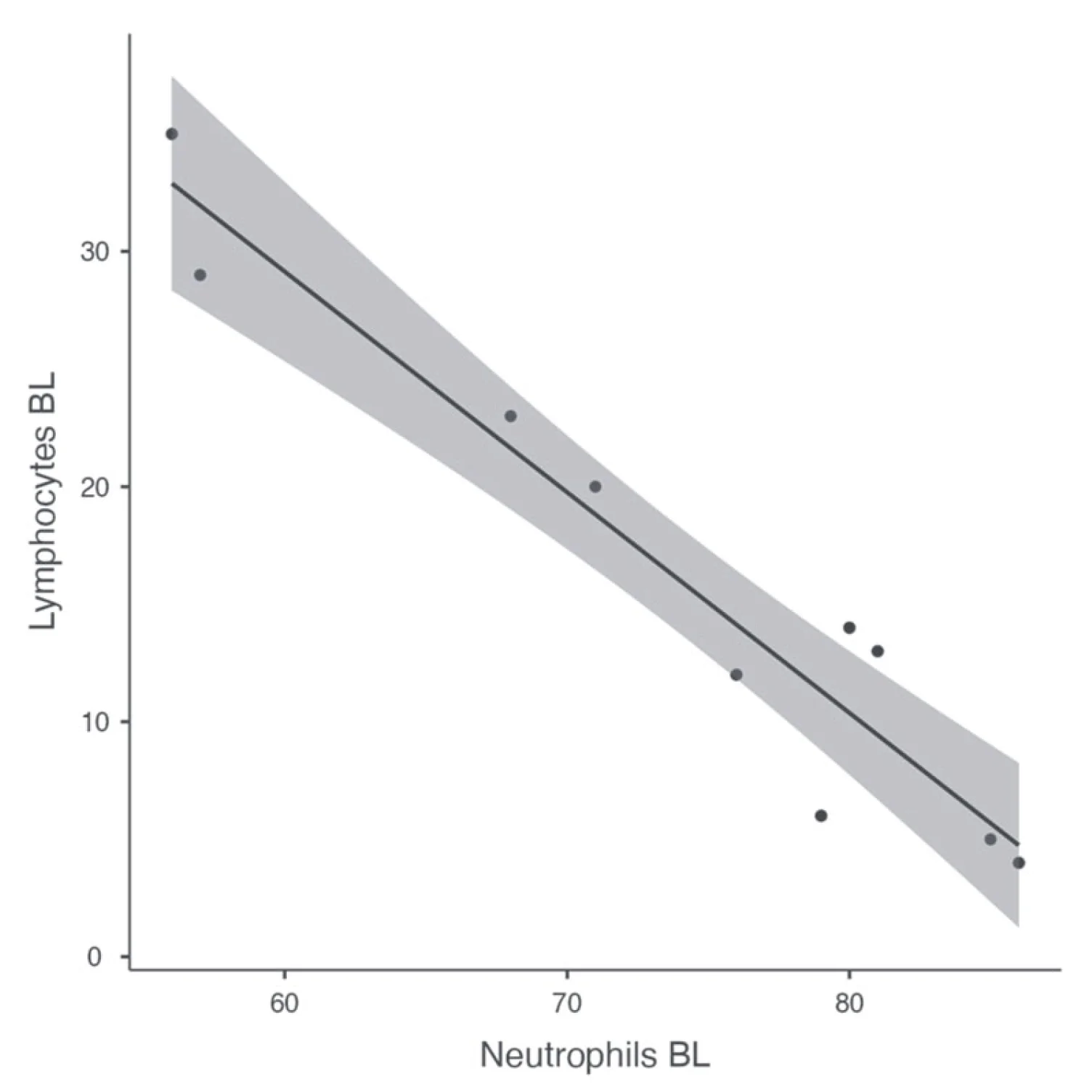
Negative correlation between lymphocytes and neutrophiles in adult females at beginning of lactation.

**Figure 4:**
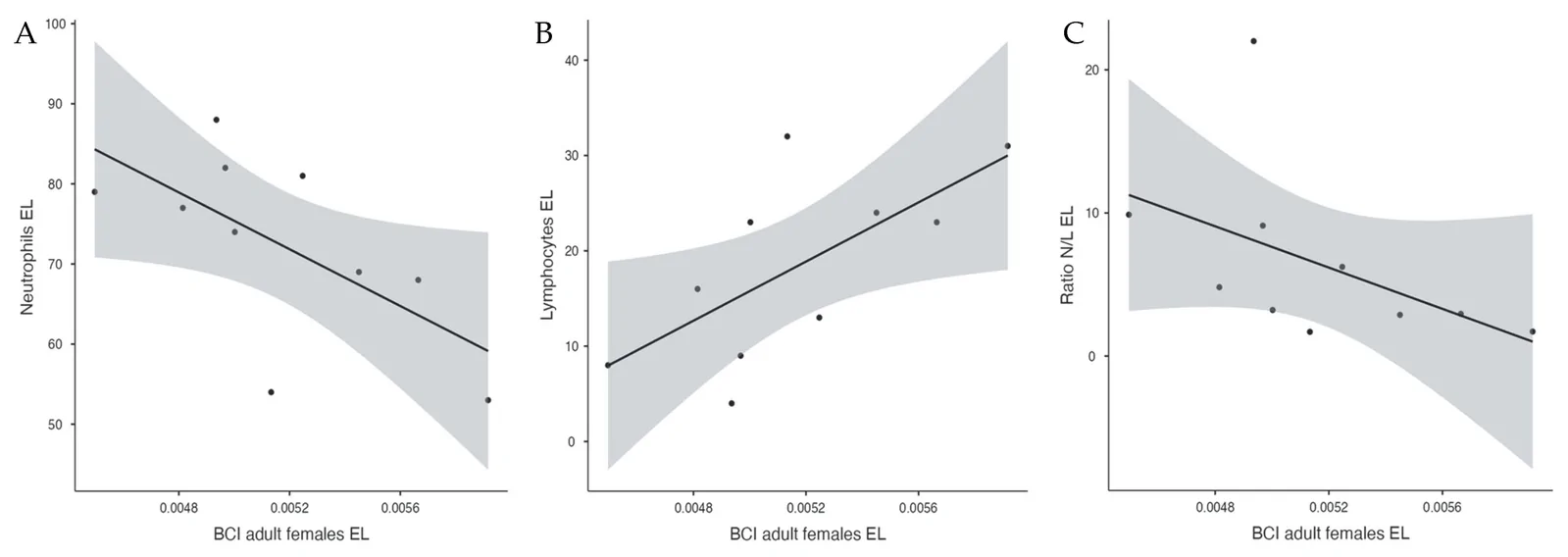
Relationships between blood cell parameters and Body Condition Index (BCI) in adult females at the end of lactation. (**A**) Negative correlation between neutrophils and BCI. (**B**) Positive correlation between lymphocytes and BCI. (**C**) Negative correlation between the N/L ratio and BCI.

**Figure 5:**
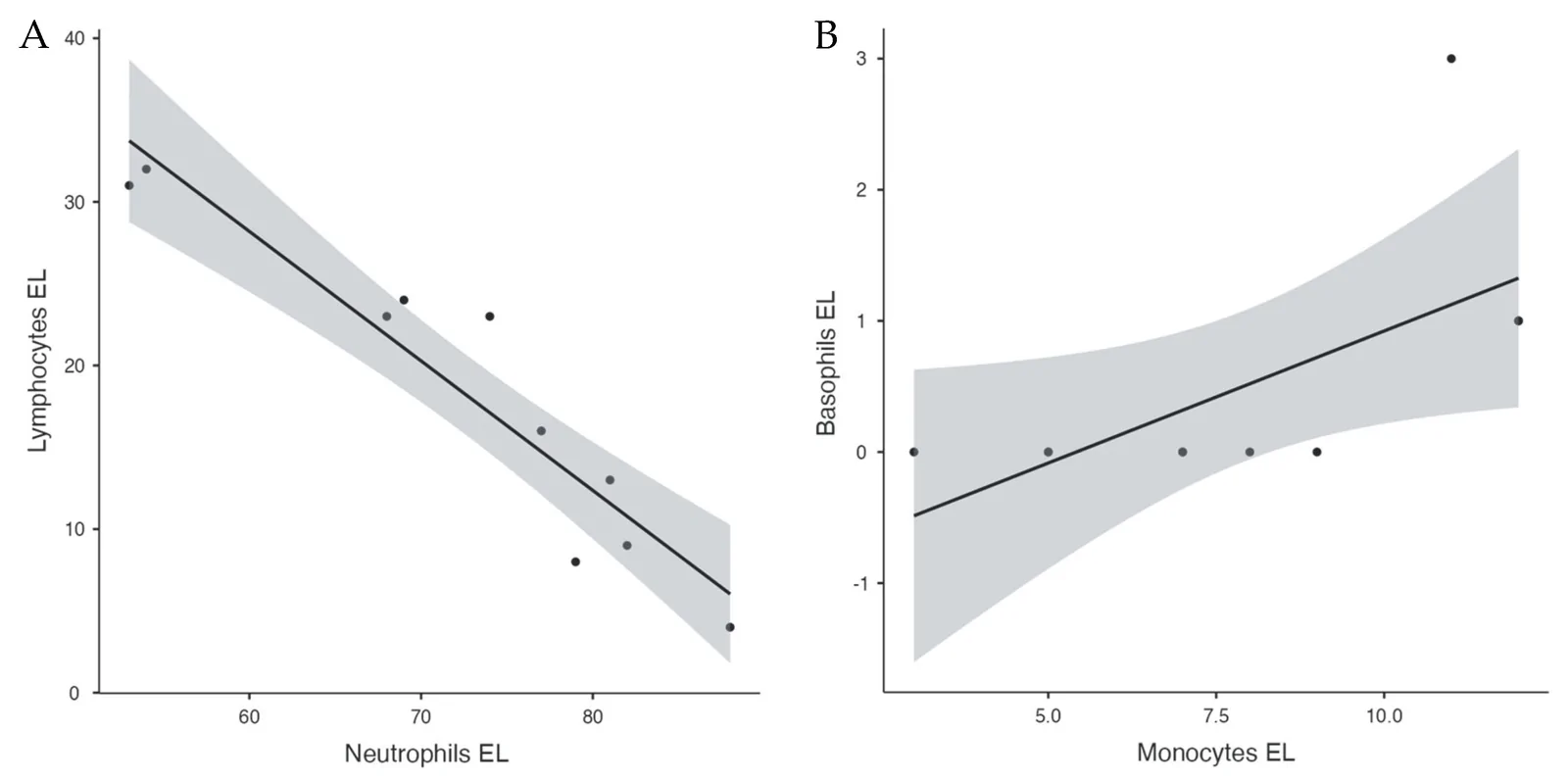
Correlations between different leukocyte types in adult females at the end of lactation: (**A**) negative correlation between lymphocytes and neutrophils; and (**B**) positive correlation between basophils and monocytes.

**Figure 6:**
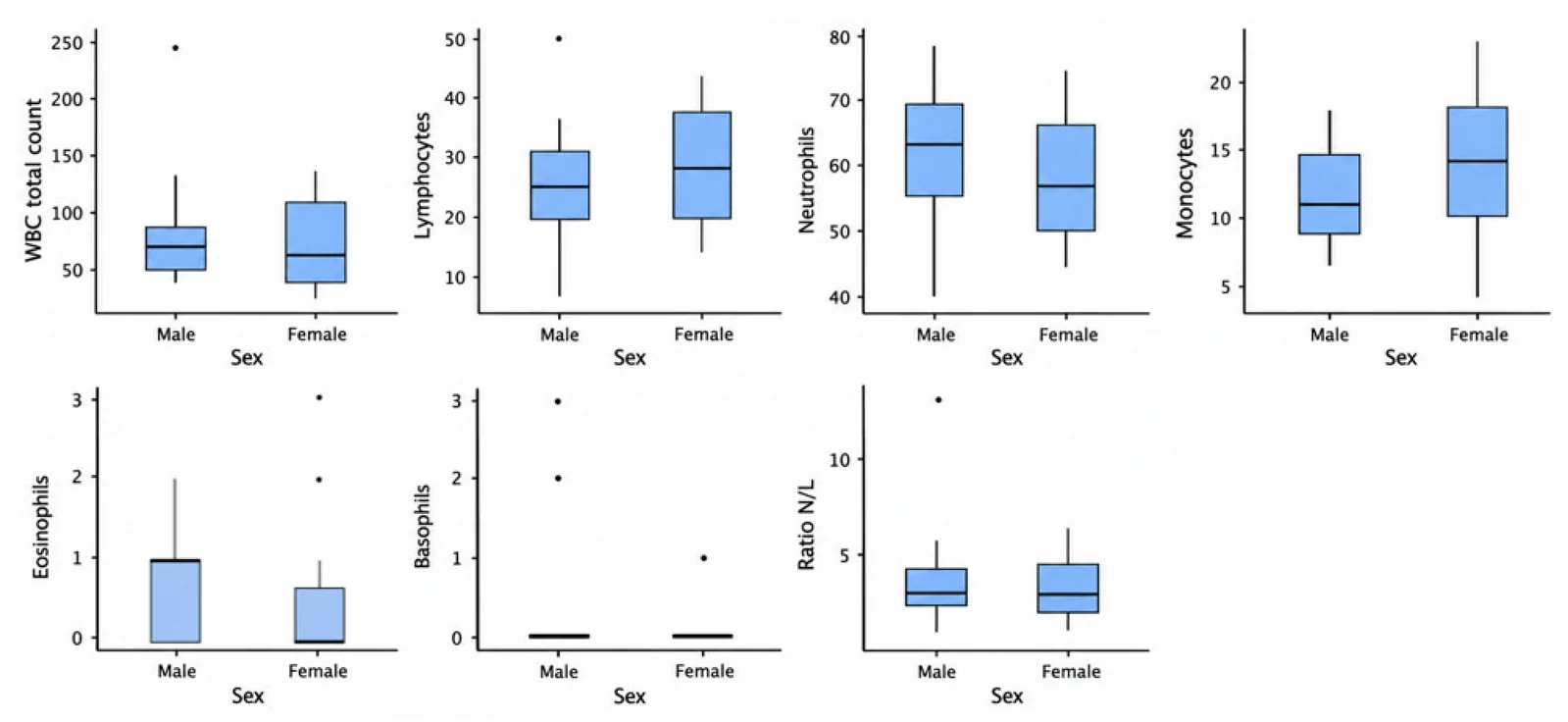
Boxplots for hematological variables comparison according to pups’ sex.

**Table 1:** Descriptive statistics for hematological parameters by age class.

	Age Class	Mean	Median	Min–Max
Total WBC count (WBC/10^4^RBC)	Adult Female	64.02 ± 50.56	47.50	6–282
	Pup	81.47 ± 48.80	72.50	24–248
Neutrophils (relative%)	Adult Female	72.97 ± 10.73	75.00	53–91
	Pup	59.78 ± 11.05	59.00	38–79
Lymphocytes (relative%)	Adult Female	17.34 ± 9.20	15.00	4–35
	Pup	26.91 ± 10.26	25.50	6–51
Monocytes (relative%)	Adult Female	8.74 ± 3.89	9	1–18
	Pup	12.56 ± 5.37	12	3–23
Eosinophils (relative%)	Adult Female	0.63 ± 0.94	0	0–3
	Pup	0.53 ± 0.80	0	0–3
Basophils (relative%)	Adult Female	0.32 ± 0.74	0	0–3
	Pup	0.22 ± 0.66	0	0–3
N/L ratio	Adult Female	6.53 ± 5.33	5.26	1.51–22
	Pup	2.88 ± 2.27	2.29	0.75–13.17

**Table 2:** Hematological Parameters by Age Class.

Parameter	Adult Female Blood Samples (*n* = 38)	Pup Blood Samples (*n* = 32)	Test	Statistic	*p*-Value	Effect Size
Total WBC count (WBC/10^4^RBC)	47.50 [29.30–83.80]	72.50 [42.00–113.00]	MWU	*U* = 446	0.057	*r_c_* = −0.25
Neutrophils	72.97 ± 10.73	59.78 ± 11.05	*t*-test	*t*(68) = 5.06	**<0.001**	d = 1.21
Lymphocytes	17.34 ± 9.20	26.91 ± 10.26	*t*-test	*t*(68) = −4.11	**<0.001**	d = −0.99
Monocytes	9.00 [7.00–11.80]	12.00 [8.50–17.00]	MWU	*U* = 370	0.005	*r_c_* = −0.39
Eosinophils	0.00 [0.00–1.00]	0.00 [0.00–1.00]	MWU	*U* = 587	0.781	*r_c_* = −0.01
Basophils	0.00 [0.00–0.00]	0.00 [0.00–0.00]	MWU	*U* = 559	0.383	*r_c_* = −0.06
Ratio N/L	5.26 [2.76–8.05]	2.29 [1.48–3.79]	MWU	*U* = 273	**<0.001**	*r_c_* = 0.55

Data are presented as mean ± SD for parametric tests and median [IQR] for non-parametric tests. The analysis includes (*n* = 38) adult female blood samples and (*n* = 32) pup blood samples. Significant *p*-values (<0.05) are in bold. Effect sizes: Cohen’s d (small: 0.2, medium: 0.5, large: 0.8); rank-biserial r (small: 0.1, medium: 0.3, large: 0.5). MWU: Mann–Whitney U test.

**Table 3:** Coefficients for correlation between hematological and biometric variables in adult females at beginning and end of lactation (*n* = 10).

	Adult Female BCI	
	Beginning of lactation	End of lactation
WBC count	*r*(8) = 0.180 [−0.507, 0.727]	*r*(8) = 0.445 [−0.257, 0.839]
	*p* = 0.619	*p* = 0.197
Neutrophils	*r*(8) = −0.180 [−0.727, 0.507]	*r*(8) = −0.640 [−0.905, −0.017]
	*p* = 0.619	***p* = 0.046**
Lymphocytes	*r*(8) = 0.202 [−0.940, 0.738	*r*(8) = 0.669 [0.068, 0.914]
	*p* = 0.576	***p* = 0.034**
Monocytes	*r*(8) = 0.086 [−0.575, 0.679]	*r*(8) = 0.330 [−0.378, 0.795]
	*p* = 0.814	*p* = 0.352
Eosinophils	*r_s_*(8) = −0.437 [−0.847, 0.298]	*r_s_*(8) = 0.473 [0.261, 0.860]
	*p* = 0.207	*p* = 0.167
Basophils	*r_s_*(8) = −0.58 [−0.898, −0.137]	*r_s_*(8) = −0.013 [−0.637, 0.622]
	*p* = 0.873	*p* = 0.972
Ratio N/L	*r*(8) = −0.099 [−0.686, 0.566]	*r_s_*(8) = −0.721 [−0.940, −0.078]
	*p* = 0.786	***p* = 0.024**
Pup ICC	*r*(8) = −0.337 [−0.797, 0.371]	*r*(8) = −0.349 [−0.802, 0.360]
	*p* = 0.341	*p* = 0.322

In bold are the values showing significant differences. Values in square brackets […] represent the 95% confidence intervals (95% CI) for the correlation coefficients. Pearson correlation was used for normally distributed variables (*r*) and Spearman rank correlation for non-normal variables (*r_s_*).

**Table 4:** Descriptives statistics for pups’ hematological parameter by sex.

	Sexe	Mean ± SD	Median [Q1–Q3]	Min–Max
WBC total count (n°WBC/10^4^)	Male	84.46 ± 61.63	75 [47.50–86]	34–248
	Female	74.67 ± 40.91	66 [39.25–114.5]	24–142
Neutrophils	Male	61.73 ± 11.73	63 [54.50–69]	38–79
	Female	57.61 ± 10.68	56 [48.75–65.5]	43–76
Lymphocytes	Male	25.82 ± 11.44	25 [19.50–30.50]	6–51
	Female	28.50 ± 10.14	28 [19.50–37.75]	13–44
Monocytes	Male	11.27 ± 3.85	10 [8–14]	7–18
	Female	13.33 ± 5.66	13.50 [9.25–17.750]	3–23
Eosinophils	Male	0.73 ± 0.79	1 [0–1]	0–2
	Female	0.44 ± 0.86	0 [0–75]	0–3
Basophils	Male	0.45 ± 1.04	0 [0–0]	0–3
	Female	0.11 ± 0.32	0 [0–0]	0–1
Ratio N/L	Male	3.44 ± 3.38	2.50 [1.90–3.42]	0.75–13.17
	Female	2.48 ± 1.48	1.98 [1.35–3.72]	1.06–5.77

**Table 5:** Mean values of the WBC differential counts measured in adult females in this study and those reported in other studies on pinnipeds.

	*Mirounga leonina*		*Mirounga angustirostris*		*Phoca vitulina richardii*	*Zalophus californianus*
	Present Study	(Melrose et al., 1995) [38]	(Yochem et al., 2008) * [15]	(Roletto, 1993) [29]	(Roletto, 1993) [29]	(Roletto, 1993) [29]
Neutrophils (%)	72.97 ± 10.73	63 ± 9	70.64	69 ± 9	63 ± 9	62 ± 11
Banded neutrophils (%)	-	-	-	2 ± 4	0 ± 1	2 ± 3
Lymphocytes (%)	17.34. ± 9.3	17 ± 7	15.94	23 ± 8	26 ± 8	30 ± 11
Monocytes (%)	8.74 ± 3.90	5 ± 2	7.29	4 ± 3	7 ± 4	4 ± 4
Eosinophils (%)	0.63 ± 0.95	15 ± 9	6.52	2 ± 2	3 ± 3	2 ± 2
Basophils (%)	0.32 ± 0.75	-	0	0 ± 0	0 ± 0	0 ± 0
N/L ratio	6.53 ± 5.34	-	-	-	-	-

* = Values in absolute numbers converted to percentages.

## Data Availability

The original contributions presented in this study are included in the article. Further inquiries can be directed to the corresponding authors.

## Appendix A

### Appendix A.1. Normality and Homogeneity Tests for Comparisons of Hematological Parameters Between Adult Females (n = 38) and Pups (n = 32)

**Table A1:** Normality test (Shapiro–Wilk).

	W	*p*
WBC total count	0.839	<0.001
Neutrophils	0.972	0.111
Lymphocytes	0.972	0.125
Monocytes	0.984	0.487
Eosinophils	0.727	<0.001
Basophils	0.488	<0.001
N/L Ratio	0.821	<0.001

**Table A2:** Homogeneity of variances test (Levene’s test).

	F	ddl	ddl2	*p*
WBC total count	6.77 × 10^−7^	1	68	0.999
Neutrophiles	0.02184	1	68	0.883
Lymphocytes	0.00511	1	68	0.943
Monocytes	5.24160	1	68	0.025
Eosinophiles	0.72336	1	68	0.398
Basophiles	0.81059	1	68	0.371
Ratio N/L	14.08214	1	68	<0.001

### Appendix A.2. Comparison Between WBC Parameters of Adult Females at the Beginning and at the End of Lactation

**Table A3:** Descriptive statistics for WBC profiles in adult females at the beginning and at the end of lactation.

	Mean ± SD	Median [Q1–Q3]	Min–Max
WBC total count BL	33.00 ± 21.68	32.00 [17.00–42.750]	6–80
WBC total count EL	52.90 ± 25.022	44.00 [32.00–77.50]	27–90
Neutrophils BL	73.90 ± 10.73	77.50 [68.75–80.75]	56–86
Neutrophils EL	72.50 ± 11.66	75.50 [68.25–80.50]	53–88
Lymphocytes BL	16.10 ± 10.48	13.50 [7.50–22.25]	4–35
Lymphocytes EL	18.30 ± 9.75	19.50 [10.00–23.75]	4–32
Monocytes BL	9.30 ± 3.40	9.50 [8.25–11.5]	3–14
Monocytes EL	7.90 ± 3.11	7.50 [5.50–10.5]	3–12
Eosinophils BL	0.60 ± 0.97	0.00 [0.00–1.00]	0–3
Eosinophils EL	0.80 ± 1.14	0.00 [0.00–1.75]	0–3
Basophils BL	0.10 ± 0.32	0.00 [0.00–0.00]	0–1
Basophils EL	0.50 ± 0.97	0.00 [0.00–0.75]	0–3
Ratio N/L BL	8.00 ± 6.86	5.97	1.60–21.50
Ratio N/L EL	6.45 ± 6.18	4.01	1.69–22.00

BL = beginning of lactation, EL = end of lactation.

**Table A4:** Paired comparison for WBC profiles in adult females at the beginning and at the end of lactation.

			Statistics	*p*-Value	Effect Size
WBC total count BL	WBC total count EL	*t* test	t(9) = −2.513	**0.033**	d = −0.795
Neutrophils BL	Neutrophils EL	*t* test	t(9) = 0.610	0.557	d = 0.193
Lymphocytes BL	Lymphocytes EL	*t* test	t(9) = −1.074	0.311	d = −0.340
Monocytes BL	Monocytes EL	*t* test	t(9) = 0.899	0.392	d = 0.284
Eosinophils BL	Eosinophils EL	*t* test	t(9) = −0.408	0.693	d = −0.129
Basophils BL	Basophils EL	Wilcoxon W	2.00	0.345	r = −0.600
Ratio N/L BL	Ratio N/L EL	*t* test	t(9) = 1.284	0.231	d = 0.406

Significant *p*-values (<0.05) are in bold.

**Table A5:** Normality test (Shapiro–Wilk) for hematological variables in adult females at the beginning (BL) and at the end of lactation (EL).

			W	*p*
WBC total count BL	-	WBC total count EL	0.979	0.957
Neutrophils BL	-	Neutrophils EL	0.922	0.376
Lymphocytes BL	-	Lymphocytes EL	0.930	0.445
Monocytes BL	-	Monocytes EL	0.892	0.176
Eosinophils BL	-	Eosinophils EL	0.912	0.292
Basophils BL	-	Basophils EL	0.775	0.007
Ratio N/L BL	-	Ratio N/L EL	0.871	0.103

### Appendix A.3. Relationship of Biometric Variables and WBC Parameters

**Table A6:** Descriptive statistics for biometric variables at beginning (BL) and end (EL) of lactation (*n* = 10).

	Mean ± SD	Median	Min − Max
BCI adult females BL	7.36 × 10^−3^ ± 7.12 × 10^−4^	7.53 × 10^–3^	6.38 × 10^−3^ − 8.71 × 10^−3^
BCI adult females EL	05.16 × 10^−3^ ± 4.21 × 10^−4^	5.07 × 10^−3^	4.50 × 10^−3^ − 5.92 × 10^−3^

**Table A7:** Normality test (Shapiro–Wilk) for adult females’ BCI at the beginning (BL) and the end (EL) of lactation (*n* = 20).

	W	*p*
BCI adult females BL	0.938	0.535
BCI adult females EL	0.973	0.917

**Table A8:** Normality test for WBC profiles in adult females at the beginning of lactation (*n* = 20).

	W	*p*
WBC total count BL	0.927	0.422
Neutrophils BL	0.893	0.185
Lymphocytes BL	0.931	0.460
Monocytes BL	0.953	0.710
Eosinophils BL	0.678	<0.001
Basophils BL	0.366	<0.001
Ratio N/L BL	0.844	0.050

**Table A9:** Normality test for adult females’ WBC profiles at end of lactation (*n* = 20).

	W	*p*
WBC total count EL	0.858	0.073
Neutrophils EL	0.914	0.309
Lymphocytes EL	0.939	0.543
Monocytes EL	0.940	0.553
Eosinophils EL	0.740	0.003
Basophils EL	0.603	<0.001
Ratio N/L EL	0.758	0.004

**Table A10:** Correlations between total adult females’ WBC parameters and their BCI (*n* = 38).

	BCI Adult Female
WBC total count	r_s_(36) = −0.26
	*p* = 0.12
Neutrophils	r_s_(36) = 0.09
	*p* = 0.59
Lymphocytes	r_s_(36) = −0.16
	*p* = 0.35
Monocytes	r_s_(36) = 0.14
	*p* = 0.42
Eosinophils	r_s_(36)= −0.02
	*p* = 0.93
Basophils	r_s_(36) = 0.08
	*p* = 0.63
N/L ratio	r_s_(36) = 0.16
	*p* = 0.34

**Table A11:** Normality test for biometric and WBC variables in total adult female samples (*n* = 38).

	W	*p*
ICC adult female	0.926	0.015
WBC total count	0.791	<0.001
Neutrophiles	0.954	0.122
Lymphocytes	0.939	0.038
Monocytes	0.976	0.593
Eosinophiles	0.695	<0.001
Basophis	0.482	<0.001
N/L ratio	0.817	<0.001

### Appendix A.4. Normality Tests for Adults Females’ Differential Body Condition Index (dBCI) (As Maternal Investment) and Correlation Coefficients for dBCI and WBC Parameters (At End of Lactation) Relationship in Our Double-Sampled Adult Females (n = 10)

**Table A12:** Normality test for adult females’ dBCI (*n* = 10).

	W	*p*
Differential BCI	0.918	0.343

**Table A13:** Correlation coefficients for dBCI and WBC parameters in double-sampled adult females (*n* = 10).

		Differential BCI
WBC total count	Pearson’s *r*	0.363
	*df*	8
	*p*	0.302
Lymphocytes	Pearson’s *r*	−0.027
	*df*	8
	*p*	0.94
Neutrophiles	Pearson’s *r*	0.084
	*df*	8
	*p*	0.817
Monocytes	Pearson’s *r*	−0.246
	*df*	8
	*p*	0.493
Eosinophiles	Spearman’s *Rho*	0.329
	*df*	8
	*p*	0.353
Basophiles	Spearman’s *Rho*	−0.27
	*df*	8
	*p*	0.451
Ratio N/L	Spearman’s *Rho*	0.127
	*df*	8
	*p*	0.733

### Appendix A.5. Comparison of WBC Parameters Between Male Pups (n = 11) and Female Pups (n = 18)

**Table A14:** Normality test (Shapiro–Wilk).

	W	*p*
WBC total count	0.854	<0.001
Neutrophils	0.972	0.607
Lymphocytes	0.986	0.957
Monocytes	0.981	0.874
Eosinophils	0.783	<0.001
Basophils	0.601	<0.001
Ratio N/L	0.716	<0.001

**Table A15:** Homogeneity of variances test (Levene’s test).

	F	ddl	ddl2	*p*
WBC total count	0.19032	1	27	0.666
Neutrophils	0.00866	1	27	0.927
Lymphocytes	0.11483	1	27	0.737
Monocytes	2.10064	1	27	0.159
Eosinophils	0.01060	1	27	0.919
Basophils	9.62218	1	27	0.004
Ratio N/L	1.41602	1	27	0.244

**Table A16:** Group comparisons for hematological parameters between male and female pups.

		Statistics	*p*	Effect Size
WBC total count	Mann–Whitney U	U = 92.00	0.770	r = −0.07
Neutrophils	*t*-test	t(27) = 0.97	0.340	d = 0.37
Lymphocytes	*t*-test	t(27) = −0.66	0.516	d = −0.25
Monocytes	*t*-test	t(27) = −1.06	0.297	d = −0.41
Eosinophils	Mann–Whitney U	U = 74.50	0.212	r = −0.25
Basophils	Mann–Whitney U	U = 90.00	0.524	r = −0.9
Ratio N/L	Mann–Whitney U	U = 80.00	0.412	r = −0.19

**Table A17:** Normality test for pups’ BCI and their mothers’ WBC profiles (*n* = 29).

	W	*p*
Pup BCI	0.888	0.005
WBC total count	0.899	0.009
Neutrophils	0.928	0.050
Lymphocytes	0.925	0.040
Monocytes	0.982	0.886
Eosinophiles	0.657	<0.001
Basophiles	0.482	<0.001
N/L ratio	0.828	<0.001

### Appendix A.6. Normality Tests for Pups’ BCI Relationships with Mother’s and Own WBC Parameters

**Table A18:** Normality test for pup BCI and WBC profiles.

	W	*p*
Pup BCI	0.957	0.283
WBC total count	0.851	<0.001
Neutrophiles	0.971	0.583
Lymphocytes	0.985	0.941
Monocytes	0.971	0.580
Eosinophiles	0.701	<0.001
Basophiles	0.410	<0.001
N/L ratio	0.672	<0.001

**Table A19:** Correlation coefficients for pup’s BCI with their mother’s biometric variable and WBC parameters.

	WBC Total Count	Neutrophils	Lymphocytes	Monocytes	Eosinophiles	Basophiles	Ratio N/L
BCI Pup	r_s_(27) = 0.18	r(27) = −0.17	r_s_ (27) = 0.21	r(27) = −0.20	r_s_(27) = 0.20	r_s_(27) = 0.09	r_s_(27) = −0.21
	*p* = 0.35	*p* = 0.38	*p* = 0.28	*p* = 0.29	*p* = 0.31	*p* = 0.65	*p* = 0.29

**Table A20:** Correlation coefficients for pup’s BCI with their WBC parameters.

	WBC Total Count	Neutrophiles	Lymphocytes	Monocytes	Eosinophiles	Basophiles	N/L Ratio
BCI pup	r_s_(27) = 0.21	r(27) = −0.01	r(27) = 0.09	r(27) = −0.17	r_s_(27) = 0.04	r_s_(27) = −0.10	r_s_(27) = 0.06
	*p* = 0.27	*p* = 0.98	*p* = 0.63	*p* = 0.381	*p* = 0.82	*p* = 0.61	*p* = 0.76
